# Vitamin C Promotes Apoptosis and Cell Cycle Arrest in Oral Squamous Cell Carcinoma

**DOI:** 10.3389/fonc.2020.00976

**Published:** 2020-06-10

**Authors:** Jianjun Zhou, Chen Chen, Xiaoqing Chen, Yifan Fei, Lei Jiang, Guodong Wang

**Affiliations:** Department of Stomatology, Changzheng Hospital, Second Military Medical University, Shanghai, China

**Keywords:** oral squamous cell carcinoma, vitamin C, reactive oxygen species, p53, p21

## Abstract

Oral squamous cell carcinoma (OSCC) is currently ranked as the eighth most prevalent type of cancer. Despite recent advances in cancer research, the 8-year survival rate for oral squamous cell carcinoma remains only 50–60%. Therefore, markers for early detection, identification of efficient chemotherapeutic agents, and post-therapeutic monitoring are the immediate needs. With this background, this study was designed to investigate the anticancer effects of vitamin C (VC) in oral squamous cell carcinoma. Our results showed that VC had an anticancer effect on the oral squamous cell lines used in this study. VC also showed an inhibitory effect on xenograft tumors in nude mice *in vitro* and had a synergistic effect with cisplatin to induce cell apoptosis. Mechanistically, VC caused a significant increase in the levels of reactive oxygen species (ROS), which led to induced genotoxic (DNA damage) and metabolic (ATP depletion) stresses, inhibited Bcl-2 expression, and promoted Bax expression and caspase-3 cleavage. VC also caused cell cycle arrest at the G0/G1 phase in OSCC cells, which is related to the activation of tumor suppressor p53 and cyclin-dependent kinase inhibitor p21. In conclusion, VC bears considerable therapeutic potential for the treatment of oral squamous cell carcinoma.

## Introduction

Oral squamous cell carcinoma (OSCC) is the most common malignant tumor in the oral and maxillofacial region. There are about 300,000 new cases every year worldwide (1, 2). As the eighth largest malignant tumor in the world, the main causes of OSCC include smoking, drinking, and viral infection (3, 4). At present, OSCC requires comprehensive treatment involving surgery, radiotherapy, chemotherapy, and immunotherapy (5). However, despite contemporary surgical techniques and integrated therapeutic strategies, such as cisplatin, paclitaxel, and gefitinib, the overall 5-year survival rate of OSCC remains at 50–60% (5, 6). Therefore, the search for new drugs with few side effects and high efficacy is in demand in order to treat OSCC patients.

Several studies have shown that consuming foods rich in antioxidants can reduce the risk of certain chronic diseases (7–9). Therefore, there is growing interest in antioxidants found naturally in fruits, vegetables, and plants. Vitamin C (L-ascorbic acid, VC) is a water-soluble vitamin and an important natural antioxidant (10). A large number of studies have shown that VC has properties of anti-oxidation, anti-inflammation, and immunity enhancement (11–13). In the 1970s, Pauling conducted clinical trials showing that intravenous VC prolonged survival in patients with advanced cancer (14–16). However, the use of VC to treat cancer was seriously questioned after subsequent trials, in which VC was taken orally, showed no benefit to cancer patients at the Mayo Clinic (17). It was recognized that the manner in which VC was administered was a key cause of differences in the treatment outcomes for cancer patients. Intravenous VC, which was used in the initial studies, was found to produce higher plasma concentrations than oral VC, as reported in subsequent trials (18). Recently, a number of studies have found that VC has anti-tumor activity in the treatment of liver, ovarian, and glioblastoma cancer (19, 20). Moreover, when the pharmacological concentration of VC is 0.05–3.52 mg/ml, multiple cancer cell lines can be selectively killed *in vitro*, but the cytotoxicity to normal cells is much lower. This pharmacologic concentration can only be achieved by intravenous injection (19, 21). For example, VC can increase the reactive oxygen species (ROS) levels in liver cancer cells through SVCT-2 uptake and then cause DNA damage and ATP depletion, ultimately leading to the apoptosis of liver cancer cells (20). It was also found that both intravenous and intraperitoneal injection of VC induced a pharmacological concentration in the blood (up to 3.52 mg/ml), while the VC concentration in the blood remained at the physiological level when it is administered orally. Additionally, VC can enhance the activity of five commonly used chemotherapy drugs (22). These findings have renewed people's attention on the pharmacological antitumor effects of VC, but its potential function and mechanism in OSCC need to be further elucidated.

## Materials and Methods

### Cell Culture

The human OSCC-derived cell lines CAL27, SCC9, and SCC25 were purchased from ATCC (Manassas, VA, USA). All cells were grown in Dulbecco's Modified Eagle Medium (DMEM, Gibco™, Invitrogen Corp, Carlsbad, CA, USA), supplemented with 10% heat-inactivated fetal bovine serum (FBS), 100 U/ml penicillin, and 100 μg/ml streptomycin. The cells were incubated at 37°C with 5% CO_2_.

### Plate Colony Formation Assay

For the plate colony formation assay, CAL27 cells, SCC9 cells, and SCC25 cells were seeded in six-well plates, at a density of 100 cells per well, and incubated with VC (Sangon Biotech Co., Ltd., China) of different concentrations for 24 h. The cells were then cultured at 37°C for 2 weeks in full-strength growth medium without VC. The cells were fixed with 4% paraformaldehyde for ~30 min and stained with 1% crystal violet for about 15 min. The colonies were counted using Image J (National Institute of Health). Each assay was repeated three times.

### Cell Proliferation Assay

For the cell proliferation assay, the cells in the growth index phase were implanted into nine six-well plates at a concentration of 4,000 cells per well. After 24 h of incubation, DMEM was replaced with fresh media containing different concentrations of VC. After 48 and 72 h, cell viability was measured by the Cell Count Kit-8 (CCK-8) assay (Dojindo Laboratories, Kumamoto, Japan). A micro-plate reader (SpectraMax i3x, Austria) was used to measure absorbance at 450 nm. The average absorbance for each concentration of VC was calculated from six wells.

### Analysis of Morphological Alterations of Cells by Light Microscopy

The morphology of CAL27 cells, SCC9 cells, and SCC25 cells treated with different concentrations of VC for 72 h was observed with an inverted microscope (CKX31, Olympus).

### Wound Healing Assay

For the cell wound healing assay, 10^6^ CAL27 cells were seeded in six-well plates containing full-strength medium until complete fusion. Then, the surface of the plate was scraped with a 20-μl sterile pipette tip and washed twice with phosphate-buffered saline to remove residual cell debris. The remaining cells were cultured in DMEM at different concentrations of VC for 24 h. The cells were observed and photographed under an inverted microscope (CKX31, Olympus) every 4–6 h at ×100 magnification. The scratch healing rate was determined using Image J software (National Institute of Health). The experiment was repeated three times.

### Cell Invasion Assay

For the cell invasion assay, 10^5^ CAL27 cells with different VC concentrations were added to the Transwell insert chamber (Corning Life Sciences, NY, USA) with matrigel (B&D Biosciences, San Jose, CA, USA) in 200 μl of DMEM medium containing 1% FBS, and 600 μl of full-strength growth medium was loaded in the lower chamber. The plates were incubated for 24 h. Then, the cells across the membrane were fixed with 4% paraformaldehyde for 30 min, and the non-invasive cells were wiped from the upper chamber with a sterile cotton ball. The fixed cells were stained with 0.1% crystal violet for about 15 min and observed with an inverted microscope (CKX31, Olympus). The number of cells that invaded the membrane was calculated from cell counts in 10 randomly selected fields. The experiment was repeated three times.

### Apoptosis Assay

Apoptosis and cell cycle arrest are two causes of cell growth inhibition (23, 24). The apoptosis assay was performed by flow cytometry using an Annexin V-FITC/PI Apoptosis Detection Kit (BD Biosciences, USA) according to the manufacturer's protocol. The cells were seeded at a density of 7 × 105 cells per well (CAL27), 3 × 105 cells per well (SCC9), and 3 × 105 cells per well (SCC25) in six-well plates and incubated with various concentrations of VC for 48 h. The cells were then resuspended in 1X binding buffer and stained with FITC Annexin V and propidium iodide (PI). Following incubation for 15 min at 25°C in the dark, the samples were immediately analyzed by flow cytometry (CyAN ADP; Beckman-Coulter). Each experiment was repeated three times.

### Cell Cycle Assay

For the cell cycle assay, the cells were seeded at a density of 7 × 10^5^ cells per well (CAL27), 3 × 10^5^ cells per well (SCC9), and 3 × 10^5^ cells per well (SCC25) in six-well plates and incubated with various concentrations of VC for 48 h. The cells were then stained with 1 ml DNA staining solution and 10 μl permeabilization solution (MultiSciences (Lianke) Biotech Co., Ltd., China). Immediately following incubation for 30 min at 25°C in the dark, the samples were analyzed by flow cytometry (CyAN ADP; Beckman-Coulter). Each experiment was repeated three times.

### Anti-tumor Property Assay

For anti-tumor property assay, nude mice were subcutaneously inoculated with 8 × 10^6^ CAL27 cells. When the average tumor diameter reached 5 mm, the mice were randomly divided into four groups, four in each group, and treatment commenced with either an intraperitoneal injection of VC (4 g/kg, twice each day), an injection of cisplatin (DDP; 3 mg/kg, twice per week), or an injection of both. The mice were euthanized after 21 days of treatment; the volumes of the tumors were measured, and the tumor growth suppression rate was calculated.

### Assessment of ROS Formation

ROS are a by-product of the mitochondrial respiratory chain and play an important role in oxidative stress. When ROS accumulate, they can damage many cellular components, including nucleic acids, proteins, and membrane lipids, ultimately leading to cell death (25). We presumed that VC can promote the production of ROS in OSCC cells, which suggests that VC is an apoptotic stimulus and is involved in mitochondria-mediated apoptosis. The assessment of ROS formation was performed by a fluorescence microscope using a ROS assay kit (Beyotime Biotechnology, China) according to the manufacturer's protocol. Briefly, SCC9 cells were seeded in six-well plates at a density of 6 × 10^5^ cells per well and incubated with VC of different concentrations for 24 h. Dichloro-dihydro-fluorescein diacetate (DCFH-DA) was then used to detect ROS production. The cells were incubated at 37°C for 20 min in 1 ml of 5 μM DCFH-DA and visualized under a fluorescence microscope (Ponteranica, Italy).

### ATP Level Assessment

The ATP levels were assessed by a microplate reader (SpectraMax i3x, Austria) using a firefly luciferase ATP assay kit (Beyotime Biotechnology, China) according to the manufacturer's protocol. The cells were seeded at a density of 7 × 10^5^ cells per well (CAL27), 3 × 10^5^ cells per well (SCC9), and 3 × 10^5^ cells per well (SCC25) in six-well plates and incubated with various concentrations of VC for 24 h. ATP content was determined by comparison to a concurrent standard curve, normalized by protein concentrations, and expressed as nmol/mg protein.

### TEM Assay

The mitochondrion is a key structure for energy production. It participates not only in energy metabolism but also in free radical metabolism (26). Mitochondrial dysfunction can lead to some serious disorders and even cell death (26, 27). The effect of VC on the mitochondria of OSCC cells was observed by transmission electron microscopy (TEM). CAL27 cells were incubated with different concentrations of VC for 48 h. After the cells were incubated with glutaraldehyde, they were dehydrated with an increased concentration of ethanol. Then, the cells were stained with uranyl acetate and lead citrate, embedded in Epon, sliced into ultrathin sections, and visualized using a transmission electron microscope (Hitachi, Tokyo, Japan).

### Western Blot Analysis

Protein extraction was performed on CAL27 cells treated with different concentrations of VC using radioimmunoprecipitation assay buffer containing a protease inhibitor cocktail and a phosphatase inhibitor cocktail. The protein was separated by 10% SDS/PAGE gel and transferred to a polyvinylidene fluoride membrane. After blocking with BSA for 2 h, the membrane was incubated overnight at 4°C with a primary antibody, including rabbit anti-p53 (1:1,000, Abcam), anti-p21 (1:1,000, Abcam), anti-Bcl-2 (1:1,000, Abcam), anti-Bax (1:1,000, Abcam), anti-cleaved-caspase-3 (1:1,000, Abcam), and glyceraldehyde 3-phosphate dehydrogenase (1:1,000, Abcam). After washing three times with Tris-buffered saline and Tween 20 (TBS-T), the membrane was incubated with an HRP-conjugated goat anti-rabbit IgG secondary antibody (1:2,000, Abcam) for 2 h. After three washes with TBS-T, the blots were incubated with a super-signal western Pico chemiluminescent substrate and visualized using the ChemiDoc XRS system (Bio-Rad).

### Statistical Analysis

Data are presented as the mean ± standard error of the mean. The difference between two groups was determined using Student's *t*-test. Multiple-group comparisons were determined using one-way analysis of variance; *p* < 0.05 was considered statistically significant.

## Results

### Vitamin C Inhibits the Growth of OSCC Cells *in vitro*

**Figure 1:**
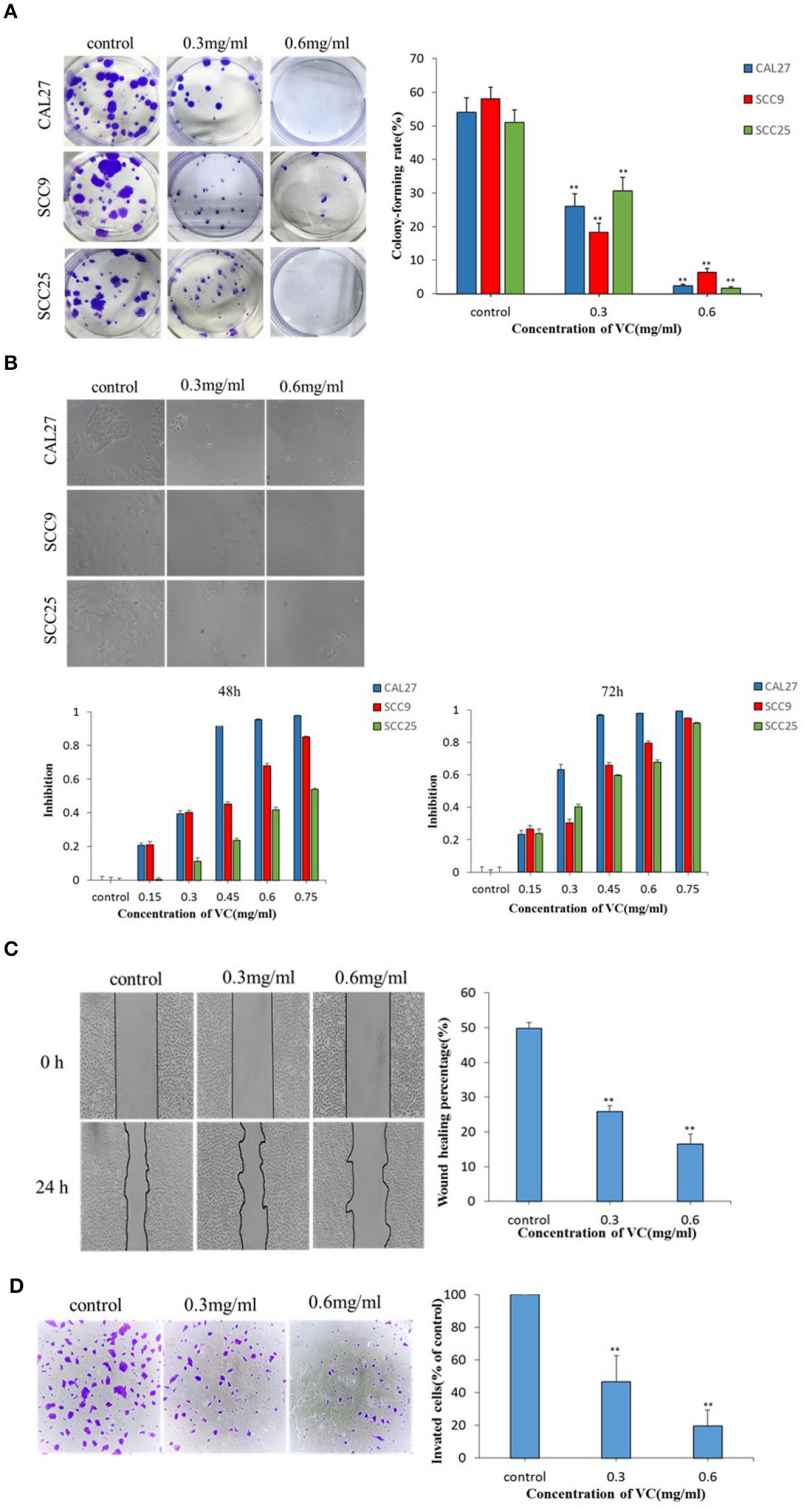
Vitamin C (VC) inhibits the growth and the migration of oral squamous cell carcinoma (OSCC) *in vitro*. **(A)** Plate colony formation assay was performed to detect the inhibitory effect of VC on the clonogenesis ability of OSCC cell lines (CAL27, SCC9, and SCC25). In a dose-dependent manner, VC reduced the ability of cells to form clones. **(B)** CAL27, SCC9, and SCC25 cells were photographed by electron microscopy after 72 h of treatment with VC of different concentrations. The normal cells appear cobblestoned, while the necrotic cells are spherical and shrunken. CCK8 assay was used to detect the cytotoxicity of VC on the proliferation of OSCC cell lines (CAL27, SCC9, and SCC25). VC decreased cell survival in a dose-dependent and time-dependent manner. **(C)** The effect of VC on the migration ability of the OSCC cell line CAL27 is detected by wound healing assay, and CAL27 cells were treated with VC of different concentrations for 24 h. **(D)** Transwell assay is used to detect the effect of VC on the invasion ability of the OSCC cell line CAL27 after treatment with different concentrations of VC for 24 h. **p < 0.01.

The effect of VC on the colony forming ability of OSCC cells was studied using a plate colony formation assay. The data showed that VC had a significant inhibitory effect on the colony forming ability of OSCC cells in a concentration-dependent manner as fewer colonies were observed for cells treated with higher concentrations of VC (Figure 1A). To observe the effect of VC on the morphology of OSCC cells, the cells were treated with VC at different concentrations for 72 h, and the cell morphology was observed by an inverted microscope. Increased VC concentration resulted in severe morphological changes in the cells, including cell shrinkage, deformation, and a decrease in the total number of cells (Figure 1B). Cell viability was measured by the cell count CCK-8 method. The results show that VC had an inhibitory effect on the proliferation of OSCC cells in a time- and concentration-dependent manner (Figure 1B) as the cell numbers decreased over time with increasing concentrations of VC. Collectively, these results suggest that VC inhibits the growth of OSCC cells *in vitro*.

### Effect of VC on the Migration and the Invasion of OSCC Cells

The effect of VC on the migration ability of CAL27 cells *in vitro* was determined by a wound healing assay. The wound healing rate of cells incubated with VC for 24 h was significantly reduced, compared to the untreated control group, in a concentration-dependent manner (Figure 1C). The effect of VC on the invasive ability of CAL27 cells *in vitro* was determined by transwell assay. The data showed that as the concentration of VC increased, the invasiveness of CAL27 cells was significantly reduced (Figure 1D). Taken together, these data indicate that VC inhibits the migration and the invasion of OSCC *in vitro*.

### VC Induced Apoptosis and G0/G1 Phase Cell Cycle Arrest in OSCC Cells

**Figure 2:**
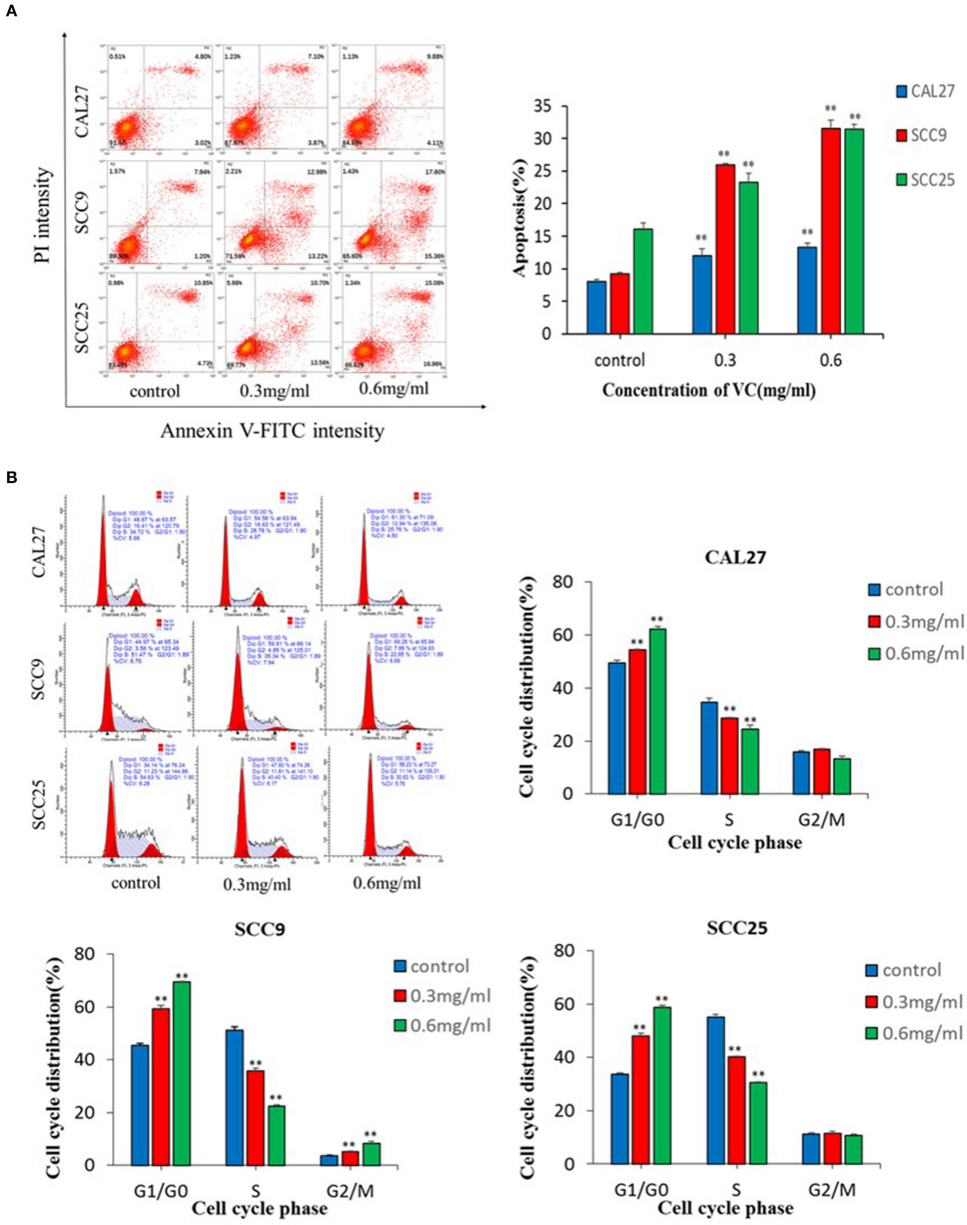
Vitamin C (VC) induced apoptosis and G0/G1 phase cell cycle arrest in oral squamous cell carcinoma cells. **(A)** Cell apoptosis was determined after 48 h of incubation with different concentrations of VC. The early apoptotic cells are located in the lower right quadrant, the late apoptotic cells are located in the upper right quadrant, and the necrotic cells are located in the upper left quadrant. **(B)** Cell cycle progression assays were performed on cells treated with different concentrations of VC for 48 h. After 48 h, VC blocked the cell cycle at the G0/G1 phase. **p < 0.01.

The data showed that VC significantly induced apoptosis in a concentration-dependent manner (Figure 2A). Induction of cell cycle arrest was detected by PI staining and flow cytometry. A concentration-dependent increase in the percentage of cells arrested in the G0/G1 cell cycle was observed following treatment with VC (Figure 2B). Thus, VC induces apoptosis and G0/G1 cell cycle arrest in OSCC cells, which may be responsible for the growth inhibition.

### VC Suppresses the Growth of OSCC in Nude Mice

**Figure 3:**
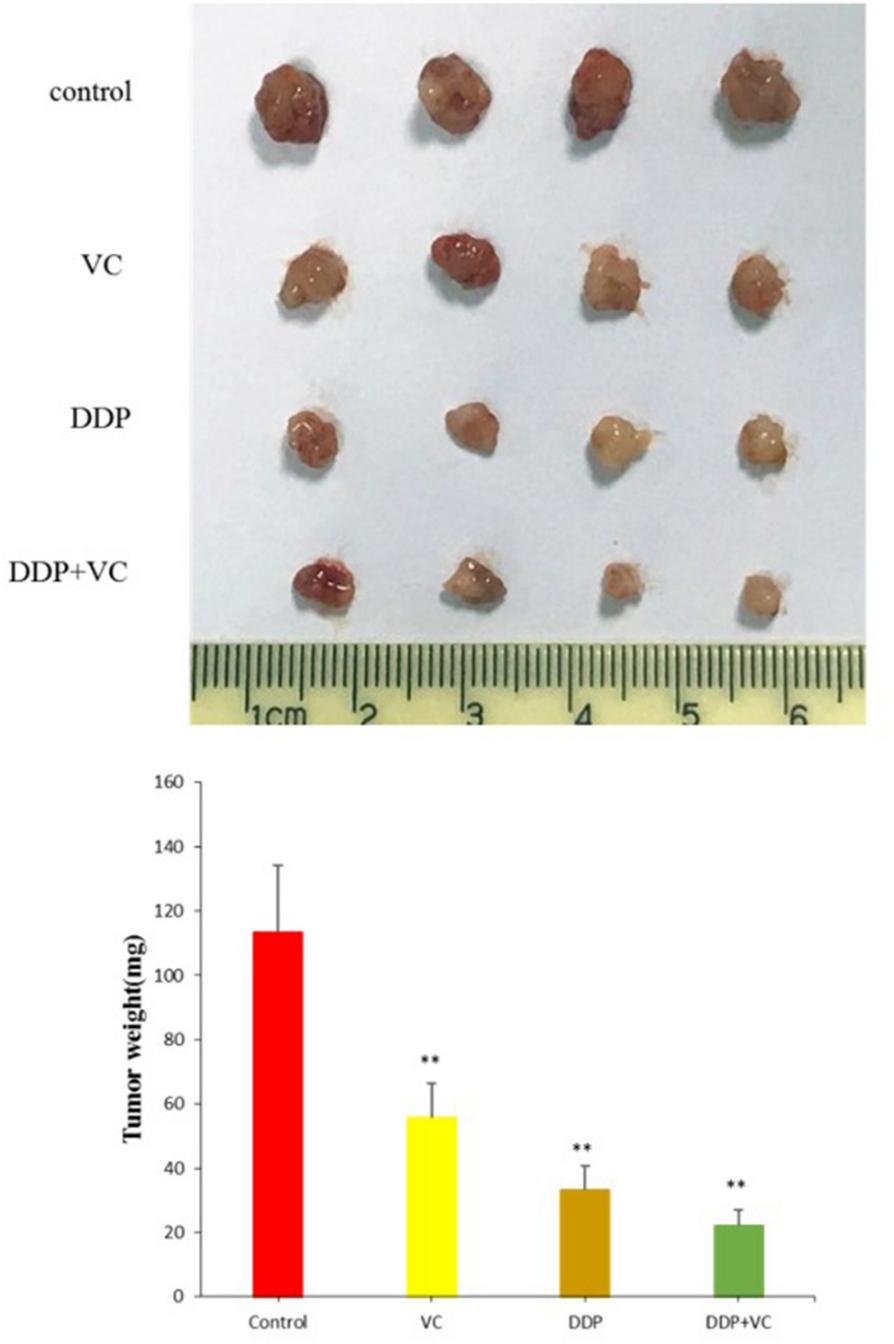
Anti-tumor growth effects of vitamin C (VC) were analyzed *in vivo*. Representative tumor images from the different treatment groups. The tumor diameter of the VC-treated group is slightly larger than that of the cisplatin (DDP)-treated group and significantly smaller than that of the saline control group. The tumor diameters of the VC + DDP combination treatment group are the smallest. **p < 0.01.

We established a subcutaneously implanted tumor model of OSCC nude mice by transplanting CAL27 cells in the axilla of nude mice. We use cisplatin to better evaluate the efficacy of VC because both VC and cisplatin are dissolved in normal saline. When the tumor diameter reached 5 mm, the mice were divided into four experimental groups, namely, the normal saline control group, the VC treatment group, the cisplatin treatment group, and the VC + cisplatin combination treatment group. VC (4 g/kg, twice per day) and DDP (3 mg/kg, twice per week) were administered continuously for 21 days. During the administration, the tumor volume of the VC group was smaller than that of the normal saline control group but slightly larger than that of the cisplatin group. The tumor volume of the VC and cisplatin combination group was the smallest (Figure 3). Hence, VC inhibits OSCC growth *in vivo* and enhances the therapeutic effect of cisplatin.

### VC Induces ROS Generation in OSCC Cells

**Figure 4:**
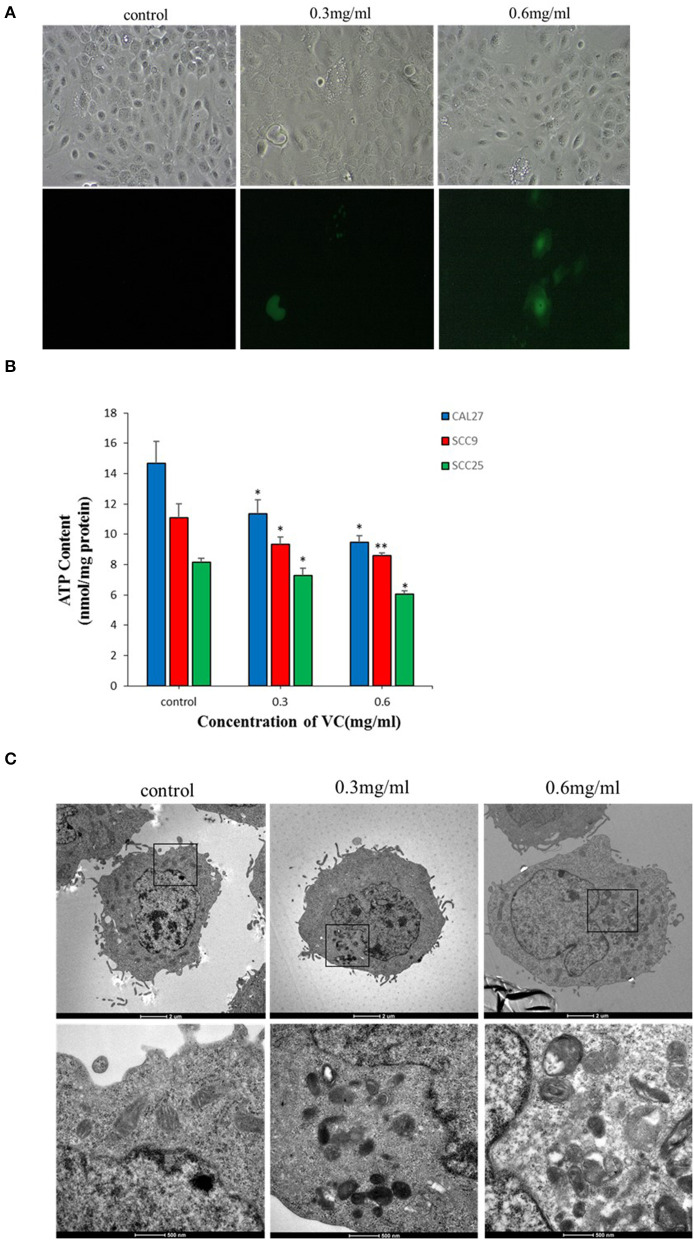
Vitamin C (VC) induces reactive oxygen species (ROS) production and causes mitochondrial damage. **(A)** The ROS levels are determined by fluorescence microscopy using dichloro-dihydro-fluorescein diacetate detection after 2 h of incubation with different concentrations of VC. VC induces ROS production in a concentration-dependent manner. **(B)** The cellular ATP levels are determined after 24 h of incubation with different concentrations of VC. The VC-treated cells exhibit decreased cellular ATP levels in a VC concentration-dependent manner. **(C)** The morphological changes of mitochondria are observed by transmission electron microscopy. Normal cell morphology and mitochondria are observed in untreated control cells. In the low-concentration-VC treatment group, the mitochondria are slightly swollen and have no obvious cristae fractures, but the vacuoles are significantly increased and a small amount of autophagous mitochondria has appeared. In the high-concentration-VC treatment group, the mitochondrial crest is broken or has disappeared, the mitochondria are swollen and vacuolated, part of the double membrane has disappeared, and a large number of autophagous mitochondria have appeared. *p < 0.05; **p < 0.01.

Intracellular ROS generation in OSCC cells was evaluated by fluorescence microscopy using DCFH-DA-based detection. After 2 h of treatment with VC, the fluorescence intensity indicating ROS generation significantly increased in VC-treated cells than in the control cells. Further, VC induced ROS production in a concentration-dependent manner (Figure 4A). Thus, the VC-mediated inhibition of OSCC progression may be due to the induced ROS generation in OSCC cells.

### VC May Cause Damage to the Mitochondria of OSCC Cells

Transmission electron microscopy revealed no obvious mitochondrial damage in the untreated control group as the organelles were structurally intact and there was almost no formation of autophagic mitochondria. In the experimental group treated with a low concentration (0.3 mg/ml) of VC, the mitochondria appeared swollen, and no obvious ridge fractures were observed, but a small amount of autophagic mitochondria appeared. In the experimental group treated with a high concentration (0.6 mg/ml) of VC, the mitochondria showed ridge rupture or disappearance, swelling, vacuolization, and disappearance of some bilayer membranes, and the appearance of a large number of autophagic mitochondria was observed, suggesting that VC may cause damage to the mitochondria (Figure 4C). The intracellular ATP content also reflects the state of the mitochondria (28). The data showed that the intracellular ATP levels were decreased in cells treated with VC for 24 h than in the untreated control group in a concentration-dependent manner (Figure 4B). Thus, the ATP analysis and the TEM results were consistent and collectively suggest that VC may cause damage to the mitochondria of OSCC cells.

### VC Induces Caspase-Dependent Apoptosis and p53/p21-Mediated Cell Cycle Arrest

**Figure 5:**
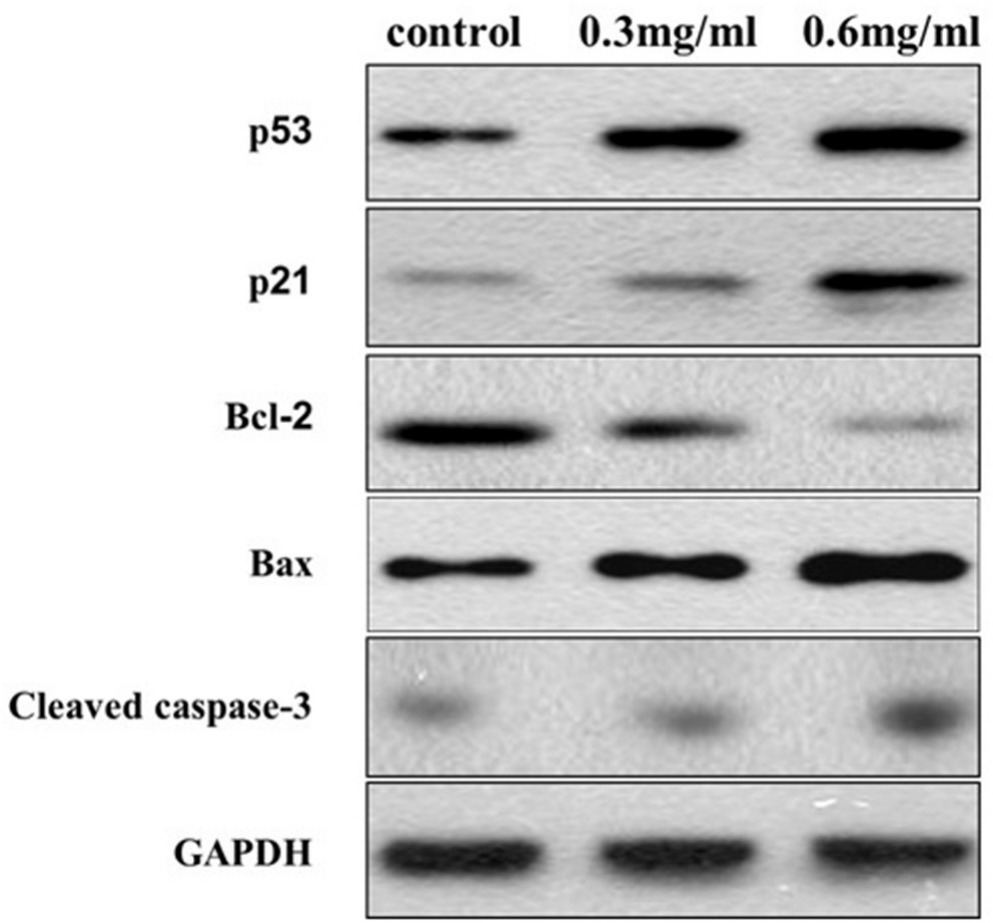
Vitamin C-induced apoptosis and cell cycle arrest may be due to the increased caspase activation and p53 and p21 expression. The expression of Bcl-2, Bax, cleaved caspase-3, p53, and p21 proteins is detected using specific antibodies against each, while glyceraldehyde 3-phosphate dehydrogenase serves as a loading control.

Western blotting was performed to further analyze the potential targets of VC in OSCC cells. We found that the protein levels of cleaved-caspase-3 and Bax, markers of apoptosis, increased with an increase in the concentration of VC, while the Bcl-2 protein levels decreased with the increased dose. We also found that VC treatment increased the expression of p53 and p21 protein levels, which are both involved in the regulation of the G0/G1 phase of the cell cycle in cancer cells (Figure 5). Thus, VC-induced apoptosis and cell cycle arrest may be due to the increased caspase activation and p53 and p21 expression.

## Discussion

Oral squamous cell carcinoma is the most common type of oral cancer, with both high incidence and high rate of malignancy (29). Primary tumor resection is the conventional treatment for OSCC, particularly since most OSCC patients are in advanced clinical stages at the time of diagnosis. Although clinical multidisciplinary collaboration and sequential treatment can improve the prognosis, the 5-year survival rate of OSCC patients is only 50–60% (30–33). Platinum chemotherapy drugs and platinum regimens are the first-line therapeutic strategies widely used in tumor chemotherapy and for OSCC treatment (34). However, platinum chemotherapeutic drugs have high toxicity and many side effects, and drug resistance may appear in patients with extensive use of platinum (35, 36). Therefore, it is necessary to search for new therapeutic drugs with high therapeutic value and few side effects.

VC is a basic dietary requirement for humans, perhaps best known for its role in preventing scurvy (10). Historically, VC has been controversial in cancer treatment. In the 1970s, Pauling found that an intravenous administration of VC to patients with advanced cancer was effective in relieving symptoms and prolonging patient survival (14–16). However, clinical trials conducted at the Mayo Clinic have found that the same dose of VC administered orally is not effective in treating cancer (17). As a result, VC was rejected as an anti-cancer drug. Subsequently, further studies found that the mode of VC administration was the most important reason for the differences in treatment outcomes as intravenous VC can produce higher plasma concentrations than VC taken orally (18). Currently, the pharmacological effects of VC have again aroused increasing interest in the field of cancer treatment.

However, few studies have explored the impact of VC on OSCC, and the potential function and the mechanism of VC treatment for OSCC remain unclear. Previous studies have found that the cytotoxicity of VC is mediated through the production of ascorbate free radicals and ROS (19, 21). Since ROS can affect a variety of cellular and molecular targets, there is currently no unified molecular mechanism reported specific to cancer cells. Relevant research reports multiple potential underlying mechanisms of ROS impact on different cancers, including mitochondrial caspase-dependent and caspase-independent apoptosis, autophagy, autolysis, ATP depletion, DNA damage, and cell cycle arrest (37–41). In OSCC, we found that ROS generated by VC caused DNA damage and ATP depletion, which in turn activated the tumor suppressor p53 and the cyclin-dependent kinase inhibitor p21, leading to G1/G0 phase cell cycle arrest and mitochondrial caspase-dependent apoptosis.

Apoptosis is a major inhibitor of cancer cell growth (38). When the mitochondrial and caspase-dependent apoptosis pathway is initiated, ATP synthesis is reduced, resulting in ATP dissipation, which in turn produces large amounts of ROS. ROS can cause a wide range of oxidative damage, including mitochondrial damage, lipid peroxidation, and DNA damage, all of which can disrupt cell growth. In addition, ROS are thought to act as second messengers that activate multiple redox-sensitive signaling cascades through interaction with Bcl-2 family proteins (42). The Bcl-2 family of proteins includes a variety of anti-apoptotic proteins (such as Bcl-2) and pro-apoptotic proteins (such as Bax), which are key factors that regulate mitochondrial membrane permeability and apoptosis (43, 44). Under normal circumstances, Bax exists in the cytoplasm and is negatively regulated by the anti-apoptotic protein Bcl-2. Therefore, the balance of Bcl-2 and Bax activity is considered to be a molecular regulator of cell fate (43). ROS can inhibit the anti-apoptotic protein Bcl-2, resulting in the activation of the pro-apoptotic protein Bax (45–47). In this study, VC increased ROS production in OSCC cells in a dose-dependent manner, leading to ATP dissipation and induction of mitochondrial damage. At the same time, VC induced the apoptosis of OSCC cells in a dose- and time-dependent manner as demonstrated by increased cell shrinkage and induced cleaved-caspase-3 in VC-treated OSCC cells. Furthermore, VC treatment increased the expression level of protein Bax and decreased the expression level of protein Bcl-2 in OSCC cells. Together these results suggest that VC induces the caspase-dependent apoptosis pathway in OSCC cells. In addition, carbon source, thymol, and icaritin have also been found to induce mitochondrial dysfunction in OSCC cells (48–50). In the future, it may be possible to combine several substances to achieve better therapeutic effects.

Cell cycle arrest is another major inhibitor of cancer cell growth (38). In our study, VC caused arrest in the G1/G0 phase of the cell cycle in OSCC cells. The tumor suppressor gene p53 plays an important role in cell cycle arrest and apoptosis, and its expression is affected by DNA damage, hypoxia, and oncogenic signals (38, 51). However, the exact mechanism by which p53 stimulates cell cycle arrest or apoptosis remains unclear (38). The p21 gene, a member of the Clp family, is a cyclin-dependent kinase inhibitor downstream of p53 gene. p53 and p21 together constitute the G1 checkpoint in the cell cycle that helps regulate the G0/G1 phase of cancer cells (52). Our results indicate that VC upregulates the expression of p53 and p21 proteins in OSCC cells, likely inhibiting cancer cells through cell cycle arrest in the G0/G1 phase.

Cancer recurrence and death for most cancer patients is due to the invasion and the metastasis of cancer cells. Thus, it is necessary to discover drugs that can inhibit both cancer cell growth and metastasis. Here we studied the effect of VC on the migration of CAL27 cells through wound healing assays and studied the effect of VC on the invasion of CAL27 cells through cell invasion assays. These assays demonstrated that VC inhibited the invasion and the migration of CAL27 cells in a dose-dependent manner. However, further research is needed to elucidate the exact mechanism by which VC inhibits cell invasion and to determine the anti-migration activity of VC in these cells.

Finally, we found a synergistic effect of VC and cisplatin on arresting the growth of OSCC cells *in vitro*. Cisplatin treatment therapy is also known to cause DNA damage, although the mechanism is different from that of VC. Cisplatin induces DNA damage through the reaction of platinum molecules with nucleophilic sites rather than through ROS (53). Thus, the combination of VC and cisplatin has the potential to induce more DNA damage to OSCC cells than either cisplatin or VC alone. Intravenous VC has also been reported to reduce the chemotherapy-related toxicity of carboplatin and paclitaxel in patients, but the specific underlying mechanism remains to be further studied.

**Figure 6:**
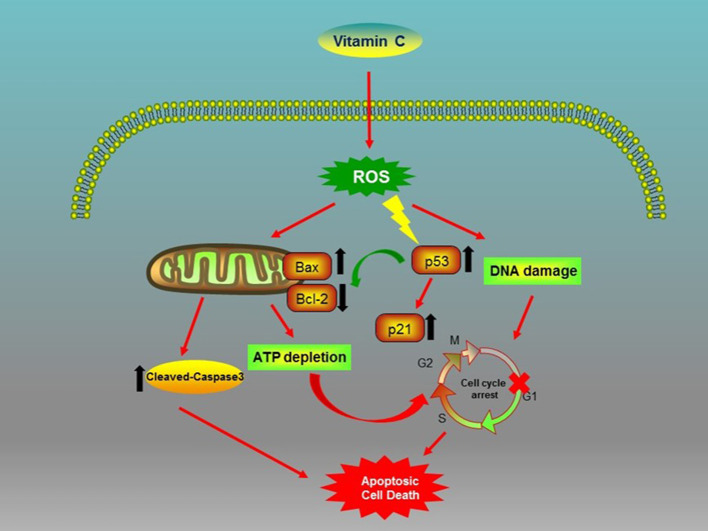
Biochemical pathways involved in the effects of vitamin C (VC) treatment on oral squamous cell carcinoma (OSCC) cells. VC inhibits the proliferation of OSCC cells *via* the induction of cell cycle arrest and apoptosis.

In conclusion, our study first shows that the VC treatment of OSCC cells generated ROS, which caused genotoxic (DNA damage) and metabolic (ATP depletion) stresses and inhibited the expression of Bcl-2 while promoting the expression of Bax and the activation of cleaved-caspase-3 (Figure 6). Consequently, VC-induced ROS activated the tumor suppressor p53 and cyclin-dependent kinase inhibitor p21, resulting in G0/G1 cell cycle arrest and mitochondrial caspase-dependent apoptosis in OSCC cells (Figure 6). Second, we discovered the potential of VC *in vitro* to resist the metastasis and the invasion of OSCC cells and the synergistic effect of VC and cisplatin on killing OSCC cells *in vitro*. Collectively, the results of our study provide support for the development of VC as a therapeutic agent for treating OSCC.

## Data Availability Statement

All datasets generated for this study are included in the article/supplementary material.

## Ethics Statement

The animal study was reviewed and approved by the Ethics Committee for Medical Research, Second Military Medical University.

## Author Contributions

JZ and CC designed and performed research, collected and analyzed data, and wrote the manuscript, XC and YF performed research and analyzed data. GW and LJ designed research, analyzed data, and wrote the manuscript. All authors contributed to the article and approved the submitted version.

## Conflict of Interest

The authors declare that the research was conducted in the absence of any commercial or financial relationships that could be construed as a potential conflict of interest.

## Abbreviations

DDP: cisplatin.
